# MCPIP1 Inhibits Hepatic Stellate Cell Activation in Autocrine and Paracrine Manners, Preventing Liver Fibrosis

**DOI:** 10.1016/j.jcmgh.2024.01.021

**Published:** 2024-02-03

**Authors:** Natalia Pydyn, Anna Ferenc, Katarzyna Trzos, Ewelina Pospiech, Mateusz Wilamowski, Olga Mucha, Piotr Major, Justyna Kadluczka, Pedro M. Rodrigues, Jesus M. Banales, Jose M. Herranz, Matias A. Avila, Tomasz Hutsch, Piotr Malczak, Dorota Radkowiak, Andrzej Budzynski, Jolanta Jura, Jerzy Kotlinowski

**Affiliations:** 1 Jagiellonian University, Faculty of Biochemistry, Biophysics and Biotechnology, Department of General Biochemistry, Krakow, Poland; 2 Jagiellonian University, Doctoral School of Exact and Natural Sciences, Krakow, Poland; 3 Malopolska Centre of Biotechnology, Jagiellonian University, Krakow, Poland; 4 Jagiellonian University Medical College, 2nd Department of General Surgery, Krakow, Poland; 5 Department of Liver and Gastrointestinal Diseases, Biodonostia Health Research Institute-Donostia University Hospital, University of the Basque Country (UPV/EHU), San Sebastian, Spain; 6 National Institute for the Study of Liver and Gastrointestinal Diseases (CIBERehd, "Instituto de Salud Carlos III"), San Sebastian-Donostia, Spain; 7 IKERBASQUE, Basque Foundation for Science, Bilbao, Spain; 8 Department of Biochemistry and Genetics, School of Sciences, University of Navarra, Pamplona, Spain; 9 Centro de Investigación Biomédica en Red de Enfermedades Hepáticas y Digestivas (CIBERehd), Carlos III National Institute of Health, Madrid, Spain; 10 Hepatology Program, Liver Unit, Instituto de Investigación de Navarra (IdisNA), Clínica Universidad de Navarra and Centro de Investigación Médica Aplicada (CIMA), Universidad de Navarra, Pamplona, Spain; 11 Department of Pathology and Veterinary Diagnostics, Institute of Veterinary Medicine, Warsaw University of Life Sciences, Warsaw, Poland; 12 Veterinary Diagnostic Laboratory ALAB Bioscience, Warsaw, Poland

**Keywords:** MCPIP1, Liver, Fibrosis, HSCs, Inflammation

## Abstract

**Background & Aims:**

Hepatic fibrosis is characterized by enhanced deposition of extracellular matrix (ECM), which results from the wound healing response to chronic, repeated injury of any etiology. Upon injury, hepatic stellate cells (HSCs) activate and secrete ECM proteins, forming scar tissue, which leads to liver dysfunction. Monocyte-chemoattractant protein-induced protein 1 (MCPIP1) possesses anti-inflammatory activity, and its overexpression reduces liver injury in septic mice. In addition, mice with liver-specific deletion of *Zc3h12a* develop features of primary biliary cholangitis. In this study, we investigated the role of MCPIP1 in liver fibrosis and HSC activation.

**Methods:**

We analyzed MCPIP1 levels in patients’ fibrotic livers and hepatic cells isolated from fibrotic murine livers. In vitro experiments were conducted on primary HSCs, cholangiocytes, hepatocytes, and LX-2 cells with MCPIP1 overexpression or silencing.

**Results:**

MCPIP1 levels are induced in patients’ fibrotic livers compared with their nonfibrotic counterparts. Murine models of fibrosis revealed that its level is increased in HSCs and hepatocytes. Moreover, hepatocytes with Mcpip1 deletion trigger HSC activation via the release of connective tissue growth factor. Overexpression of MCPIP1 in LX-2 cells inhibits their activation through the regulation of *TGFB1* expression, and this phenotype is reversed upon MCPIP1 silencing.

**Conclusions:**

We demonstrated that MCPIP1 is induced in human fibrotic livers and regulates the activation of HSCs in both autocrine and paracrine manners. Our results indicate that MCPIP1 could have a potential role in the development of liver fibrosis.

## Graphical abstract

**Figure:**
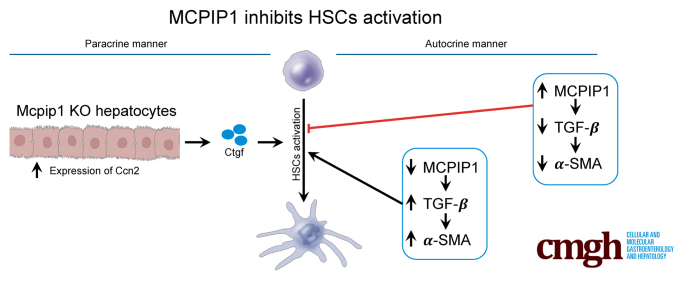

**Summary**

Mcpip1 is an endoribonuclease that targets proinflammatory transcripts. Our discoveries prove that it inhibits hepatic stellate cell activation in both autocrine and paracrine manners. Overexpression of Mcpip1 protein in HSCs inhibits their activation, and on the other hand, knockout of Mcpip1 in hepatocytes results in enhanced release of Ctgf leading to HSCs activation.

Hepatic fibrosis is characterized by enhanced deposition of extracellular matrix (ECM), which results from the wound healing response of the liver to a chronic and repeated injury of any etiology.^1^ Liver fibrosis is mostly associated with nonalcoholic steatohepatitis (NASH); however, hepatic fibrosis is also a common feature of hepatitis C virus infection, alcoholic liver disease, cholestatic disorders, and autoimmune hepatitis.^1^, ^2^, ^3^ The end-stage consequence of long-term untreated fibrosis is liver cirrhosis, which over time requires liver transplantation or can lead to patient’s death.^4^

The liver consists of parenchymal cells (60%–70%), hepatocytes, and nonparenchymal cells (30%–40%), which include endothelial cells, Kupffer cells, hepatic stellate cells (HSCs), cholangiocytes, and intrahepatic lymphocytes.^5^ The most important cells involved in fibrogenesis are HSCs. In a quiescent state, these cells reside in the space of Disse, interposed between liver sinusoidal endothelial cells (LSECs) and hepatocytes, where they account for vitamin A storage in the liver.^1^^,^^6^ During chronic liver injury, hepatocyte damage leads to an inflammatory reaction, and these events trigger HSC activation and transdifferentiation into myofibroblast-like cells, along with the acquisition of proinflammatory and profibrogenic properties. Activated HSCs migrate and accumulate at the sites of liver injury, where they secrete large amounts of ECM proteins and regulate their degradation.^1^ If the injury is sustained, accumulated ECM replaces damaged parenchymal cells, forming scar tissue, which disturbs liver architecture and can further result in organ dysfunction.^7^

Monocyte-chemoattractant protein-induced protein 1 (MCPIP1), encoded by the *ZC3H12A* gene, is a protein that possesses anti-inflammatory activity. This protein is an RNase that degrades a wide variety of transcripts, for example, those coding proinflammatory cytokines such as interleukin (IL) 1β,^8^ IL-6,^9^ and IL-2.^10^ Furthermore, through cooperation with USP10 and TANK proteins and subsequent deubiquitination of TRAF6 by USP10, MCPIP1 negatively regulates nuclear factor kappa B activity.^11^ *ZC3H12A* is differentially expressed in human tissues, and the highest level of MCPIP1 is found in leukocytes, but MCPIP1 is also expressed in the liver, spleen, lungs, and heart.^8^ Moreover, MCPIP1 was shown to be involved in the suppression of the inflammatory response in liver macrophages. As shown by Li et al,^12^ overexpression of MCPIP1 reduces liver injury in septic mice by inhibiting the inflammatory reaction. We have also observed that Mcpip1^fl/fl^Alb^Cre^ mice, with specific deletion of Mcpip1 in liver epithelial cells, spontaneously develop features of primary biliary cholangitis.^13^ Previous research concerning the role of MCPIP1 in silicosis, a type of pulmonary fibrosis, was performed using cellular models reflecting the molecular changes that occur in the context of this disease. On the basis of these experiments, it was shown that SiO_2_ has an impact on the increased activation of macrophages via MCPIP1. In addition, the proliferation and migration of fibroblasts, which are signaling events in the development of pulmonary fibrosis, were enhanced by MCPIP1 in this model.^14^, ^15^, ^16^ Recent studies have also shown that a lack of MCPIP1 in macrophages aggravates chronic renal inflammation and promotes the loss of tubular epithelial cells and renal fibrosis upon kidney ischemia‒reperfusion injury.^17^

In the present study, we aimed to analyze the role of MCPIP1 in liver fibrosis and the process of HSC activation. We examined MCPIP1 levels in fibrotic liver samples collected from patients. Then, we evaluated Mcpip1 expression in hepatic cells isolated from carbon tetrachloride (CCl_4_)-treated mice and in HSCs from Mcpip1^fl/fl^Alb^Cre^ mice. We also investigated the impact of MCPIP1 on the activation status of HSCs in both a paracrine and autocrine manner. Our results provide new insight into the antifibrotic role of MCPIP1.

## Results

### Development of Liver Fibrosis in Patients Results in Increased Hepatic MCPIP1 Levels or ZC3H12A Expression

**Figure 1:**
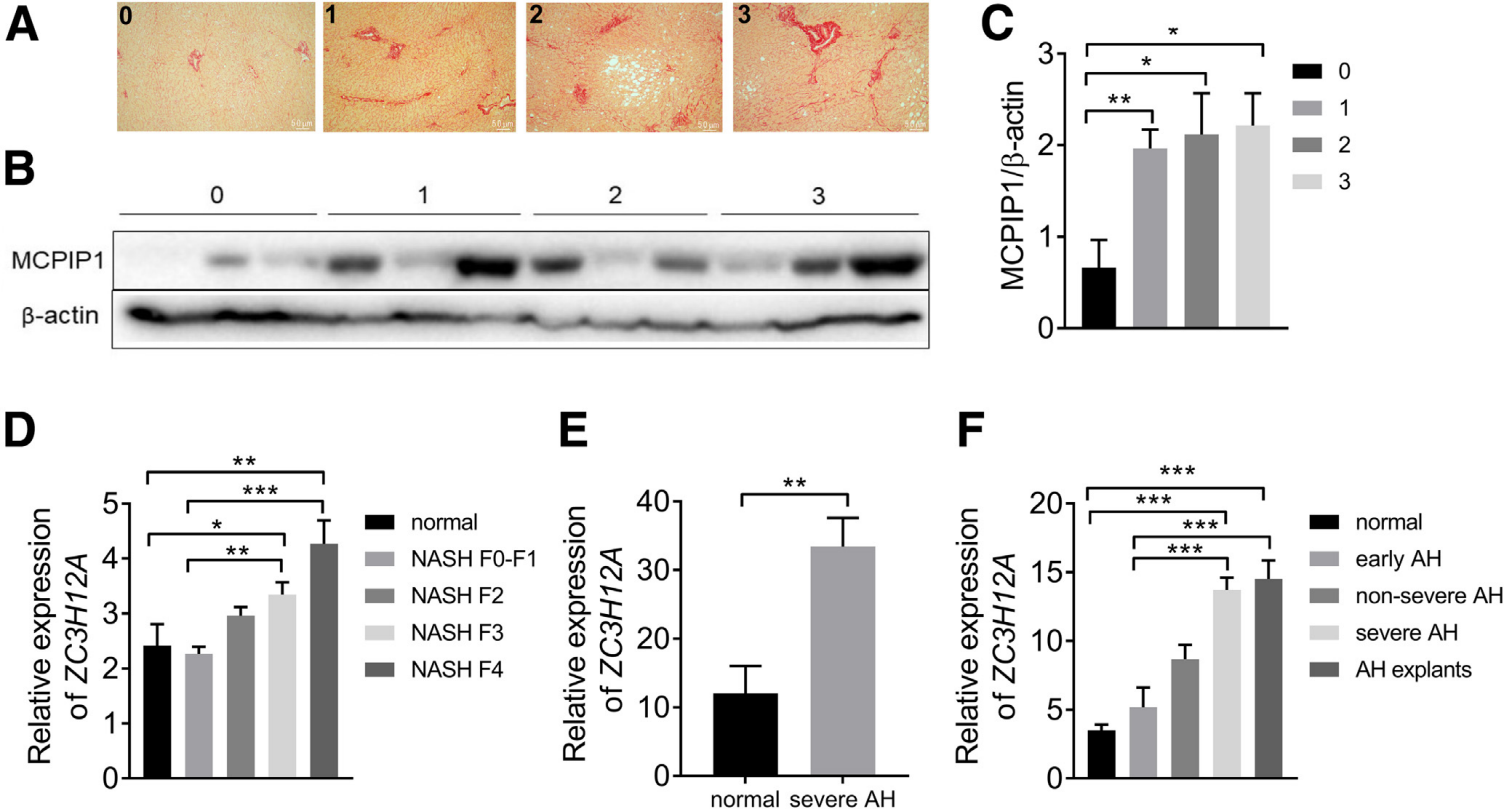
**Liver fibrosis in nonalcoholic fatty liver disease and AH patients is accompanied by an increase in MCPIP1 protein levels or *ZC3H12A* expression.** (*A*) Representative PSR staining, original magnification ×100. (*B*) Western blot analysis and (*C*) densitometric quantification of MCPIP1 levels in liver tissue (for stage F0, n = 5; for F1, n = 12; for F2, n = 10; for F3, n = 5). Expression of the *ZC3H12A* gene in livers of (*D*) patients with NASH-associated fibrosis (for normal, n = 10, for NASH F0-F1, n = 34, for NASH F2, n = 53, for NASH F3, n = 54, for NASH F4, n = 14), (*E*) (n = 5) and (*F*) patients with AH (for normal, n = 10, for early AH, n = 12, for nonsevere AH, n = 11, for severe AH, n = 18, for AH explants, n = 11). Graphs show means + SEMs; ∗*P* < .05, ∗∗*P* < .01, ∗∗∗*P* < .001.

We previously found that MCPIP1 protein levels are reduced in the livers of nonalcoholic fatty liver patients.^18^ In this study, we analyzed liver samples obtained from 32 patients who underwent surgery at the Collegium Medicum Jagiellonian University. Depending on the stage of fibrosis, we divided the patients into four (0–3) groups according to the staging score developed by Brunt et al^19^ (Figure 1*A*). To study the possible involvement of MCPIP1 in liver fibrosis, we examined its levels in these liver tissue samples. Western blot analysis revealed that MCPIP1 levels were significantly higher in liver samples from patients in groups 1 (n = 12), 2 (n = 10), and 3 (n = 5) than in those from patients in group 0 (n = 5) (Figure 1*B* and *C*). In addition, bioinformatic analysis revealed that in the cohort of NASH patients analyzed by Govaere et al,^20^ divided according to fibrosis stage, *ZC3H12A* expression was significantly induced in the livers of patients from the F3 and F4 groups compared with those of the normal liver and F0–F1 groups (Figure 1*D*). Analysis of another data set provided by Hyun et al^21^ demonstrated that the expression of *ZC3H12A* is also induced in the livers of patients suffering from severe alcoholic hepatitis (AH) (Figure 1*E*). More detailed analyses of liver samples for AH patients were performed by Argemi et al,^22^ who divided patients into early AH, nonsevere AH, and severe AH groups and included a set of explants from patients with AH who underwent urgent liver transplantation (AH explants). *ZC3H12A* expression was significantly higher in liver samples from patients with nonsevere AH, severe AH, and AH explants than in samples from normal liver (Figure 1*F*). We also observed that *ZC3H12A* expression increased with increasing disease severity (Figure 1*F*).

### Mcpip1 Levels Are Increased in Hepatocytes and Hepatic Stellate Cells in CCl_4_-induced Liver Fibrosis in Mice

**Figure 2:**
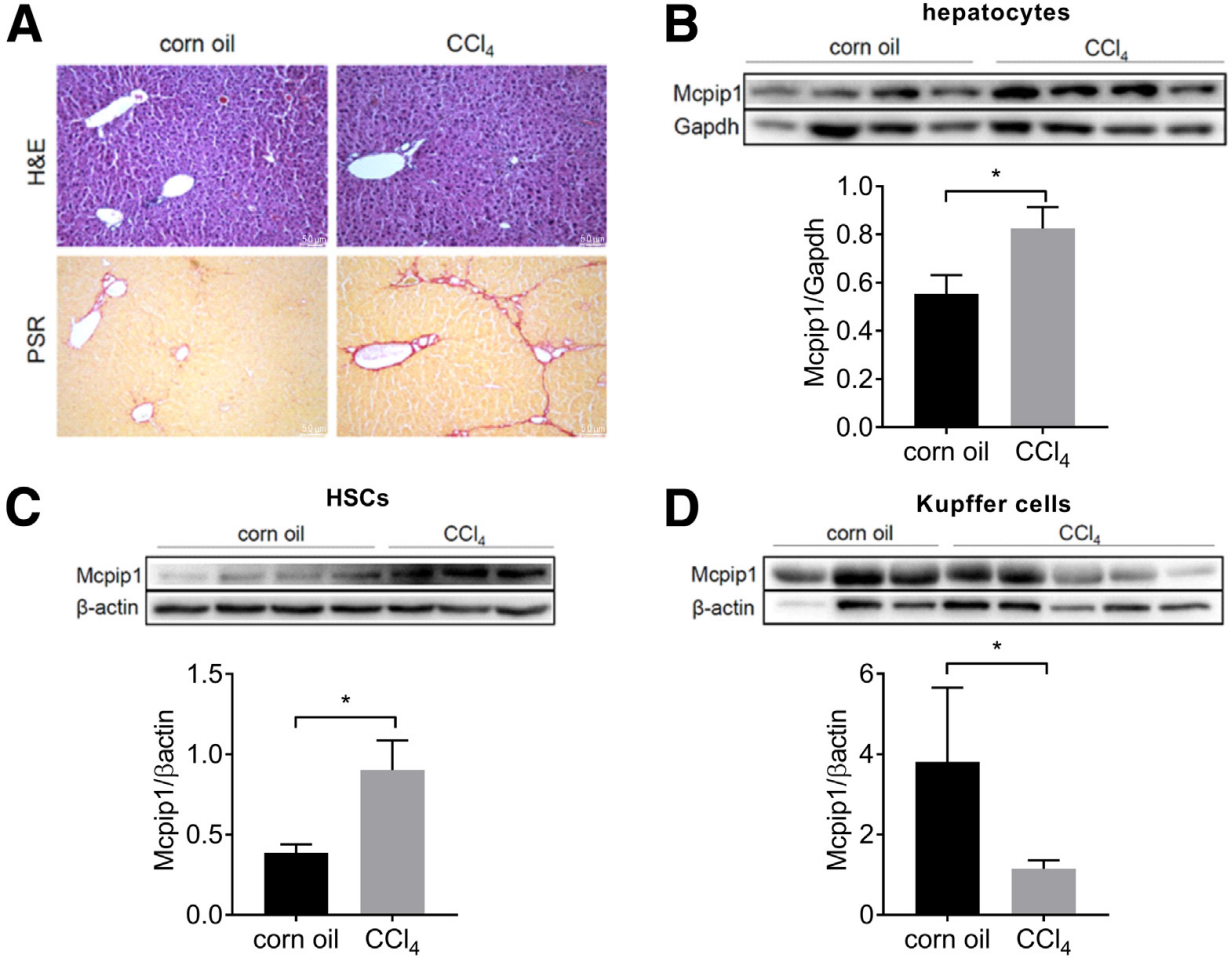
**Mcpip1 levels are increased in HSCs and hepatocytes from CCl**_**4**_**-treated mice.** (*A*) Representative H&E and PSR staining; original magnification, ×100. Western blot analysis and densitometric quantification of Mcpip1 levels in (*B*) hepatocytes, (*C*) HSCs, and (*D*) Kupffer cells. For corn oil, n = 3–4, and for CCl_4,_ n = 3–5. Graphs show means + SEMs; ∗*P* < .05.

**Figure 3:**
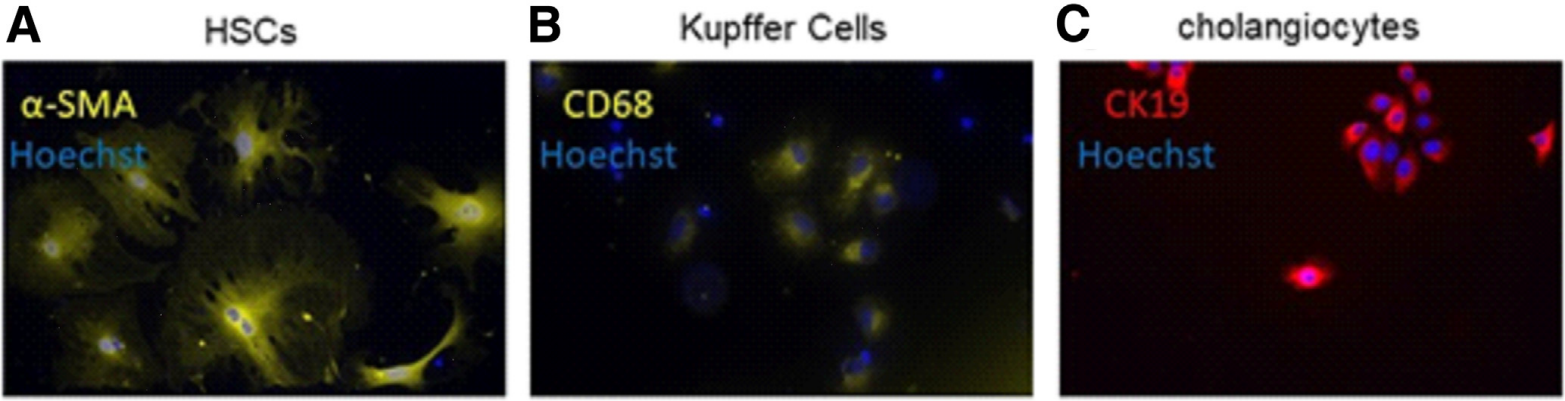
**Representative immunofluorescence staining.** Representative immunofluorescence staining of (*A*) α-SMA in HSCs, (*B*) CD68 in Kupffer cells, and (*C*) CK19 in cholangiocytes.

To elucidate which type of liver cells show induction of Mcpip1 during fibrogenesis, we induced the development of fibrosis in mice by CCl_4_ administration (Figure 2*A*). After 4 weeks of treatment, we analyzed Mcpip1 levels in hepatocytes, HSCs, and Kupffer cells isolated from control or fibrotic murine livers (Figure 3*A* and *B*). We demonstrated that treatment with CCl_4_ induced Mcpip1 levels in hepatocytes and HSCs (Figure 2*B* and *C*). In contrast, fibrosis led to decreased Mcpip1 levels in Kupffer cells (Figure 2*D*).

### Liver Fibrosis in Mcpip1^fl/fl^Alb^Cre^ Mice Is Accompanied by Mcpip1 Up-regulation in HSCs

**Figure 4:**
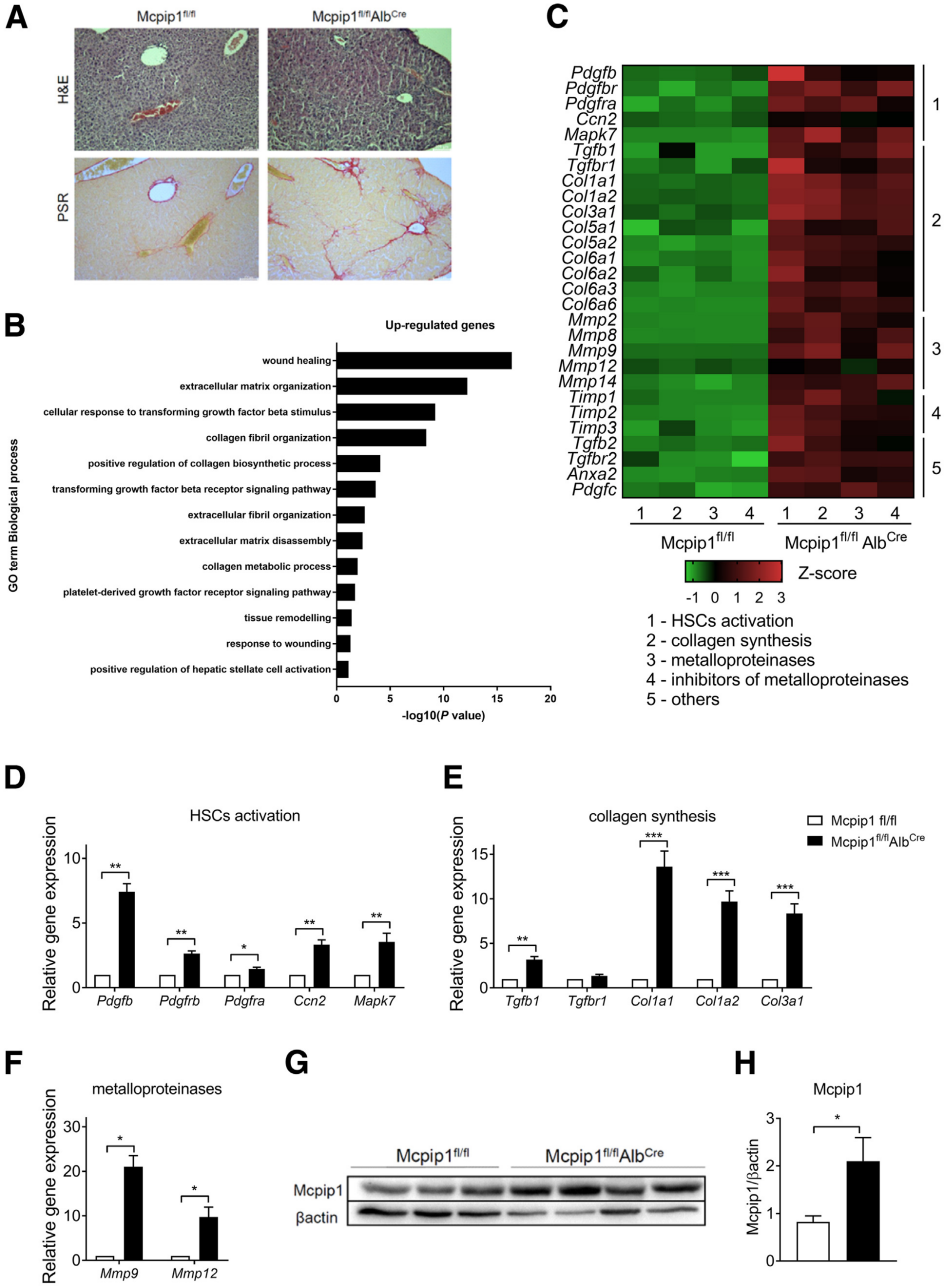
**Liver fibrosis in Mcpip1**^**fl/fl**^**Alb**^**Cre**^**mice is accompanied by induction of Mcpip1 protein in HSCs.** (*A*) Representative H&E and PSR staining; original magnification, ×100. (*B*) GO enrichment analysis of genes differentially expressed in 6-week-old Mcpip1^fl/fl^ and Mcpip1^fl/fl^Alb^Cre^ livers. Scale is −log10(*P* value) of enrichment score (*P* value < .05). (*C*) Heatmap showing differentially expressed genes selected from GO enrichment analysis; expression of genes coding for proteins involved in (*D*) HSC activation, (*E*) collagen synthesis, and (*F*) metalloproteinases. (*G*) Western blot analysis and (*H*) densitometric quantification of Mcpip1 levels in HSCs. For Mcpip1^fl/fl^, n = 3–5; for Mcpip1^fl/fl^Alb^Cre^, n = 4–5. Graphs show means + SEMs; ∗*P* < .05, ∗∗*P* < .01, ∗∗∗*P* < .001.

**Figure 5:**
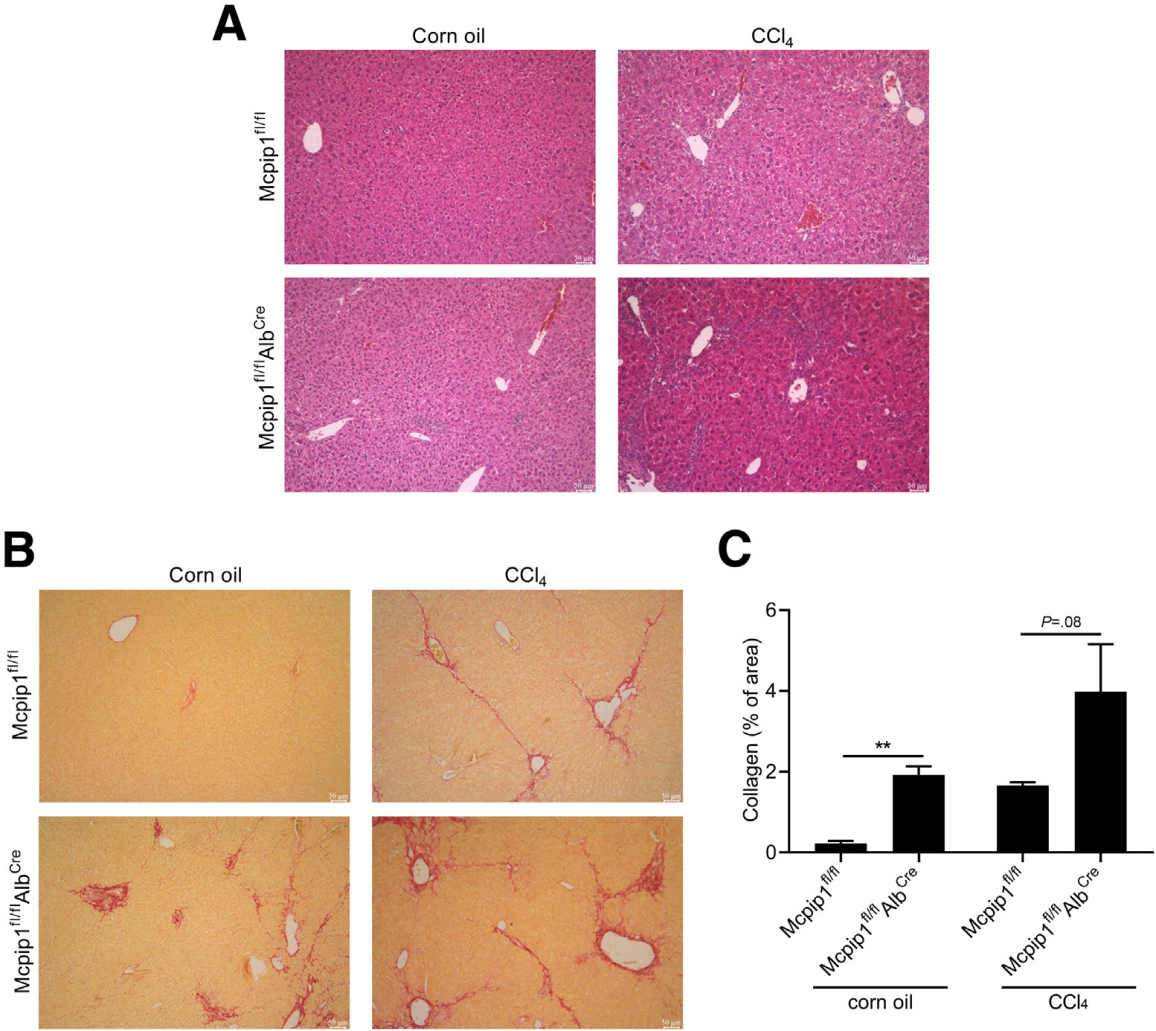
**Analysis of CCl**_**4**_**-induced liver fibrosis in Mcpip1**^**fl/fl**^**and Mcpip1**^**fl/fl**^**Alb**^**Cre**^**mice.** Representative (*A*) H&E and (*B*) PSR staining; original magnification, ×100. (*C*) Quantification of collagen area in murine livers; from each mouse right medial, right lateral, and caudate lobes were analyzed, and next mean value was calculated for each animal, n = 6. Graph shows means + SEMs; ∗∗*P* < .01.

Furthermore, to study the role of Mcpip1 in liver homeostasis, we used Mcpip1^fl/fl^Alb^Cre^ mice, which spontaneously develop liver fibrosis (Figure 4*A*). The liver transcriptome of 6-week-old Mcpip1^fl/fl^Alb^Cre^ mice was analyzed and compared with that of their respective age-matched controls, Mcpip1^fl/fl^ mice. To identify enhanced pathways associated with liver injury emerging from the lack of Mcpip1 in liver epithelial cells, we performed Gene Ontology (GO) term enrichment analysis. As shown in Figure 4*B* and *C*, up-regulated genes encoded proteins involved in ECM organization and disassembly, Tgf-β and Pdgf receptor signaling pathways, collagen biosynthetic and metabolic processes, and regulation of HSC activation. In livers from Mcpip1^fl/fl^Alb^Cre^ mice, up-regulation of genes involved in HSC activation (*Pdgfb, Pdgfbr, Pdgfra, Ccn2, Mapk7*), collagen synthesis (*Tgfb1, Tgfbr1, Col1a1, Col1a2, Col3a1*), and degradation of ECM (*Mmp9, Mmp12*) was confirmed by real-time polymerase chain reaction (PCR) (Figure 4*D–F*). In addition, HSCs isolated from the livers of both control and Mcpip1^fl/fl^Alb^Cre^ mice revealed that Mcpip1 levels were induced in cells collected from knockout animals (Figure 4*G* and *H*). Finally, control and Mcpip1^fl/fl^Alb^Cre^ animals were subjected to liver fibrosis induced by CCl_4_ treatment. Intraperitoneal injections of CCl_4_ induced liver fibrosis in all mice regardless of their genotype; collagen area increased 7 times in Mcpip1^fl/fl^ and 2 times in Mcpip1^fl/fl^Alb^Cre^ (Figure 5).

### Transforming Growth Factor Beta–Stimulated Activation of LX-2 Cells Results in Increased Levels of MCPIP1

**Figure 6:**
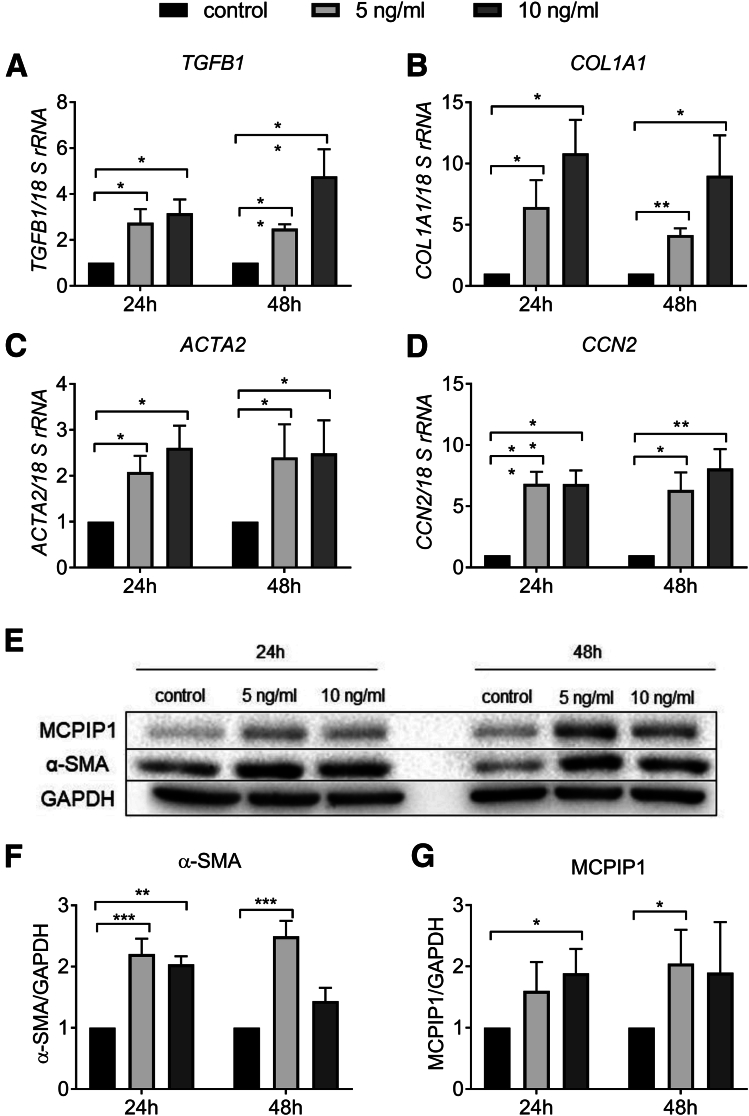
**MCPIP1 levels are increased in activated LX-2 cells.** Expression of (*A*) *TGFB1*, (*B*) *COL1A1*, (*C*) *ACTA2*, and (*D*) *CCN2* in LX-2 cells incubated with control medium or stimulated with 5 ng/mL or 10 ng/mL TGF-β for 24 and 48 hours. (*E*) Western blot analysis and densitometric quantification of (*F*) α-SMA and (*G*) MCPIP1 protein levels in LX-2 cells incubated with control medium or stimulated with 5 ng/mL or 10 ng/mL TGF-β for 24 and 48 hours, n = 5. Graphs show means + SEMs; ∗*P* < .05, ∗∗*P* < .01, ∗∗∗*P* < .001.

Because Mcpip1 is also up-regulated in HSCs isolated from fibrotic livers, we next analyzed its intrinsic role in the activation of these cells. For this purpose, we treated human hepatic stellate cell line LX-2 cells with 5 ng/mL and 10 ng/mL transforming growth factor beta (TGF-β) for 24 and 48 hours. Stimulation with TGF-β resulted in up-regulated protein expression of activation markers such as *TGFB1, COL1A1, ACTA2,* and *CCN2* and alpha-smooth muscle actin (α-SMA) (Figure 6*A–F*). We found that MCPIP1 levels were also induced in activated LX-2 cells (Figure 6*G*).

### Down-regulation of MCPIP1 Results in the Development of an Activated Phenotype in LX-2 Cells

**Figure 7:**
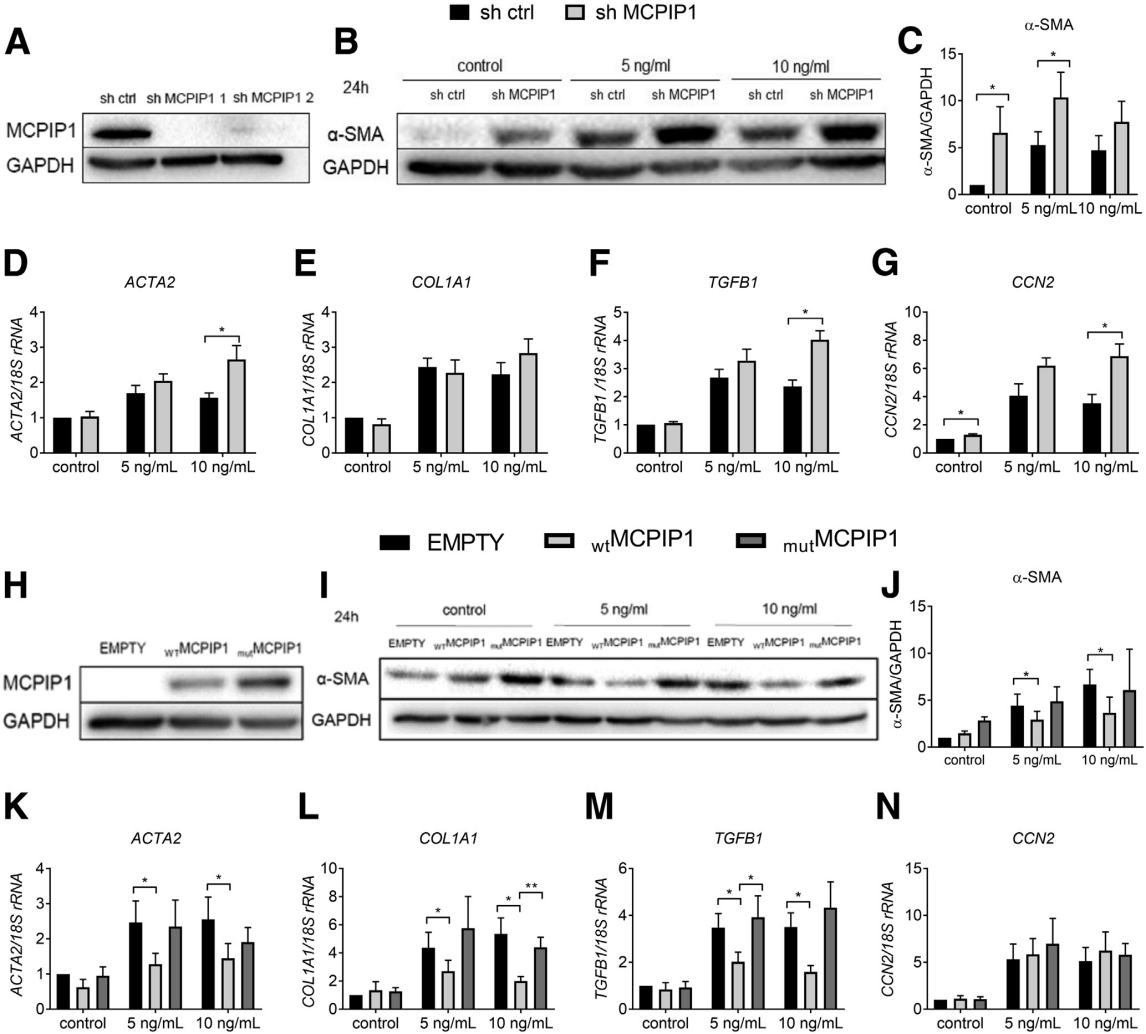
**Silencing MCPIP1 results in enhanced activation of LX-2 cells, and its RNase activity is indispensable for attenuation of LX-2 activation.** (*A*) Representative Western blot analysis of MCPIP1 silencing obtained using lentiviral vectors with 2 different sh sequences (sh MCPIP1 1 and sh MCPIP1 2) for silencing *ZC3H12A* gene expression. (*B*) Western blot analysis and (*C*) densitometric quantification of α-SMA levels in LX-2 cells incubated with control medium or stimulated with 5 ng/mL or 10 ng/mL TGF-β for 24 hours. Expression of (*D*) *ACTA2, (E*) *COL1A1, (F*) *TGFB1,* and *(G*) *CCN2* in LX-2 cells incubated with control medium or stimulated with 5 ng/mL or 10 ng/mL TGF-β for 24 hours. (*H*) Representative Western blot analysis of MCPIP1 levels in LX-2 cells transduced with pLIX EMPTY, pLIX _WT_MCPIP1, and pLIX _mut_MCPIP1 lentiviral vectors in the Tet-On system. (*I*) Western blot analysis and (*J*) densitometric quantification of α-SMA levels in LX-2 cells incubated with control medium or stimulated with 5 ng/mL or 10 ng/mL TGF-β for 24 hours. Expression of (*K*) *ACTA2, (L*) *COL1A1, (M*) *TGFB1,* and *(N*) *CCN2* in LX-2 cells incubated with control medium or stimulated with 5 ng/mL or 10 ng/mL TGF-β for 24 hours, n = 5. Graphs show means + SEMs; ∗*P* < .05; ∗∗*P* < .01.

**Figure 8:**
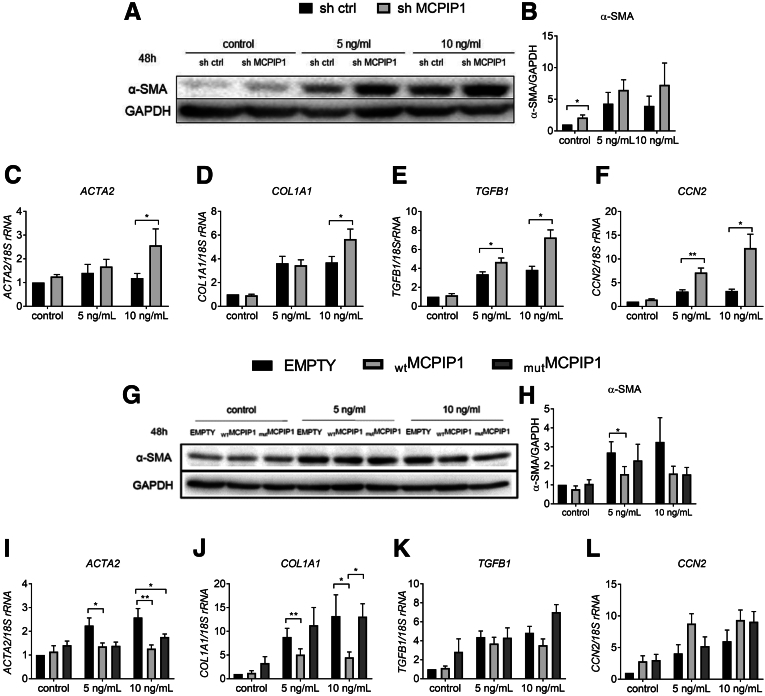
**Silencing MCPIP1 results in enhanced activation of LX-2 cells, and its RNase activity is indispensable for attenuation of LX-2 activation.** (*A*) Representative Western blot analysis and (*B*) densitometric quantification of α-SMA levels in LX-2 cells incubated with control medium or stimulated with 5 ng/mL or 10 ng/mL TGF-β for 48 hours. Expression of (*C*) *ACTA2, (D*) *COL1A1, (E*) *TGFB1,* and *(F*) *CCN2* in LX-2 cells incubated with control medium or stimulated with 5 ng/mL or 10 ng/mL TGF-β for 48 hours. (*G*) Representative Western blot analysis and (*H*) densitometric quantification of α-SMA levels in LX-2 cells incubated with control medium or stimulated with 5 ng/mL or 10 ng/mL TGF-β for 48 hours. Expression of (*I*) *ACTA2, (J*) *COL1A1, (K*) *TGFB1,* and *(L*) *CCN2* in LX-2 cells incubated with control medium or stimulated with 5 ng/mL or 10 ng/mL TGF-β for 48 hours, n = 5. Graphs show means + SEMs; ∗*P* < .05; ∗∗*P* < .01.

Because MCPIP1 levels are induced in LX-2 cells stimulated with TGF-β, to study the potential influence of MCPIP1 on the activation of HSCs, we transduced LX-2 cells with vectors encoding short hairpin (sh)RNA targeting the *ZC3H12A* gene (sh MCPIP1 1 and sh MCPIP1 2) or a control vector (sh ctrl). As shown in Figure 7*A*, both shRNA sequences were able to decrease the amount of MCPIP1 protein (Figure 7*A*). For further experiments, we decided to use the sh MCPIP1 1 sequence (shMCPIP1). Silencing MCPIP1 resulted in a significant increase in α-SMA protein levels in control cells and those treated with 5 ng/mL TGF-β (Figure 7*B* and *C*). We also discovered increased expression of *ACTA2, TGFB1,* and *CCN2* after stimulation with 10 ng/mL TGF-β for 24 hours in cells with MCPIP1 silencing (Figure 7*D–G*). Stimulation for 48 hours resulted in higher levels of α-SMA protein in the cells incubated with control medium (Figure 8*A* and *B*). Similarly, the expression of *ACTA2*, *COL1A1, TGFB1,* and *CCN2* was increased in the sh MCPIP1 LX-2 cells after stimulation with 5 and 10 ng/mL TGF-β (Figure 8*C–F*). An increase in the protein level and gene expression of these activation markers may indicate greater activation of LX-2 cells upon MCPIP1 silencing.

### Ectopic MCPIP1 Overexpression Inhibits the Activation of LX-2 Cells

To investigate whether ectopic MCPIP1 overexpression impacts the activation of HSCs, we overexpressed wild-type MCPIP1 (_WT_MCPIP1) and its enzymatically inactive form with D141N point mutation (_mut_MCPIP1) in LX-2 cells (Figure 7*H*). In accordance with our previous experiments, we found that α-SMA protein levels and the expression of *ACTA2*, *COL1A1,* and *TGFB1*, but not *CCN2,* were decreased in the _WT_MCPIP1 cells stimulated with 5 and 10 ng/mL TGF-β for 24 hours compared with the cells transduced with empty vector (EMPTY) (Figure 7*I–N*). Likewise, α-SMA protein levels were reduced in the cells with _WT_MCPIP1 ectopic expression stimulated with 5 ng/mL TGF-β for 48 hours (Figure 8*G* and *H*). In addition, after 48 hours of stimulation with 5 and 10 ng/mL TGF-β, the expression of *ACTA2* and *COL1A1*, but not *TGFB1* and *CCN2*, was diminished on _WT_MCPIP1 overexpression (Figure 8*I–L*). Importantly, no changes were detected on overexpression of enzymatically inactive _mut_MCPIP1. These observations indicate that the RNase activity of MCPIP1 is indispensable for the inhibition of LX-2 activation.

**Figure 9:**
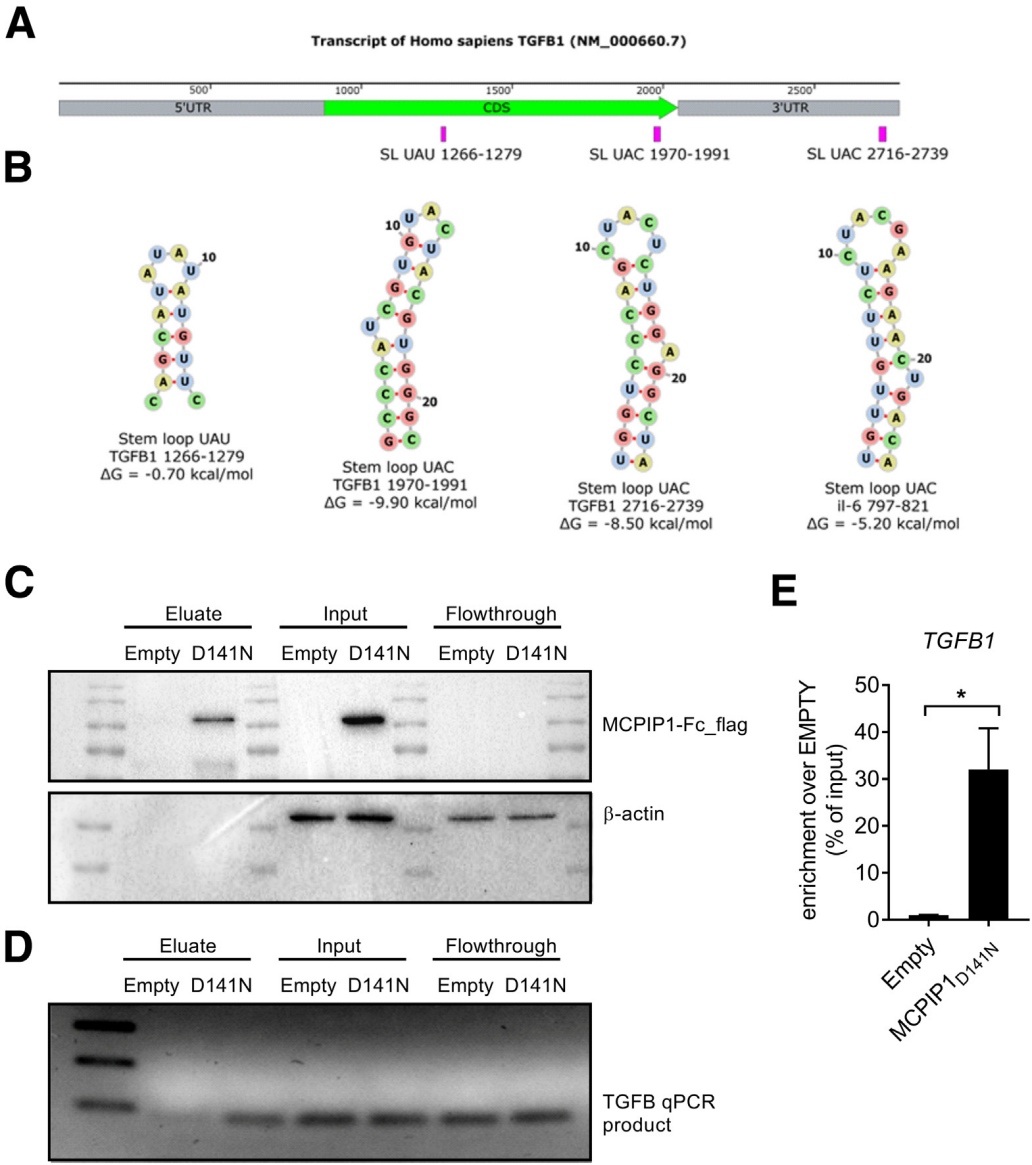
**Analysis of structural elements of *TGFB1* transcript and MCPIP1-TGFB1 mRNA interactions.** (*A* and *B*) In silico predicted hairpins in *TGFB1* mRNA that are potential targets for endonuclease degradation by MCPIP1 due to the presence of the YRY sequence in the loop of the hairpin. For structure-sequence comparison, the stem‒loop from the murine *Il6* transcript (NM_031168.2) is shown as a known motif for MCPIP1-induced cleavage. Representative results of (*C*) Western blot and (*D*) quantitative PCR performed after MCPIP1_D141N_-Fc RNA immunoprecipitation. (*E*) Results of quantitative PCR analysis of “Eluate” fractions. Enrichment of *TGFB1* mRNA expression (enrichment) over cells transfected with empty plasmid, n = 3. Graph shows means + SEMs; ∗*P* < .05.

The MCPIP1 endonuclease has been shown to be selective for the binding of stem‒loop structures that contain a pyrimidine-purine-pyrimidine (YRY) motif in sequences of single-stranded regions of RNA hairpins.^23^ Therefore, we analyzed the global folding of *TGFB1* mRNA for the presence of hairpins with YRY sequences in a loop (Figure 9*A* and *B*). This analysis revealed that 3 stem‒loop structures with UAU or UAC sequences in the loops are possibly folded in *TGFB1* mRNA. Next, to analyze whether MCPIP1 protein can bind *TGFB1* mRNA, a protein-RNA immunoprecipitation experiment was performed. As demonstrated in Figure 9, eluate fraction obtained from cells overexpressing MCPIP1_D141N_-Fc was enriched in *TGFB1* mRNA in comparison with empty sample (Figure 9*C–E*).

### HSC Activation Is Induced in a Paracrine Manner by Hepatocytes From Mcpip1^fl/fl^Alb^Cre^ Mice

**Figure 10:**
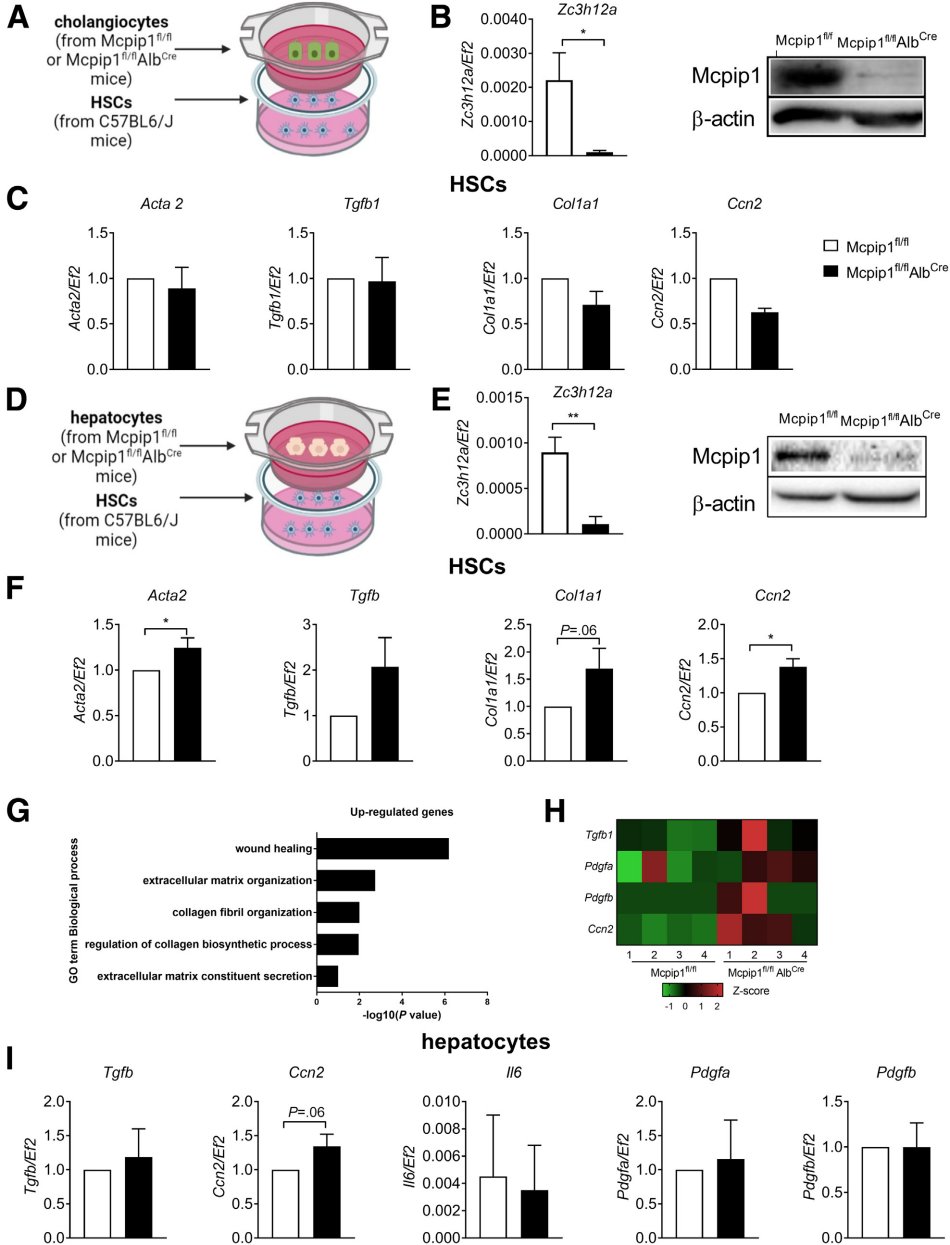
**Primary hepatocytes from Mcpip1**^**fl/fl**^**Alb**^**Cre**^**mice induce HSCsactivation in a paracrine manner.** (*A*) Schematic representation of cholangiocyte and HSC co-culture in Transwell inserts. (*B*) Expression of *Zc3h12a* gene and Western blot analysis of Mcpip1 protein levels in cholangiocytes. (*C*) Expression of *Acta2, Tgfb1, Col1a1,* and *Ccn2* in HSCs co-cultured with cholangiocytes from Mcpip1^fl/fl^ or Mcpip1^fl/fl^Alb^Cre^ mice. (*D*) Schematic representation of hepatocyte and HSC co-culture in Transwell inserts. (*E*) Expression of *Zc3h12a* gene and Western blot analysis of Mcpip1 protein levels in hepatocytes. (*F*) Expression of *Acta2, Tgfb1, Col1a1,* and *Ccn2* in HSCs co-cultured with hepatocytes from Mcpip1^fl/fl^ or Mcpip1^fl/fl^Alb^Cre^ mice. (*G*) GO enrichment analysis of selected genes differentially expressed in 6-week-old Mcpip1^fl/fl^ and Mcpip1^fl/fl^Alb^Cre^ primary hepatocytes. Scale is −log10(*P* value) of the enrichment score (*P* value < .05). (*H*) Heatmap showing differentially expressed genes selected from GO enrichment analysis. (*I*) Expression of *Tgfb1*, *Ccn2, Il6, Pdgfa,* and *Pdgfb* in hepatocytes from Mcpip1^fl/fl^ or Mcpip1^fl/fl^Alb^Cre^ mice co-cultured with HSCs, n = 3. Graphs show means + SEMs; ∗*P* < .05; ∗∗*P* < .01. Created with BioRender.com.

**Figure 11:**
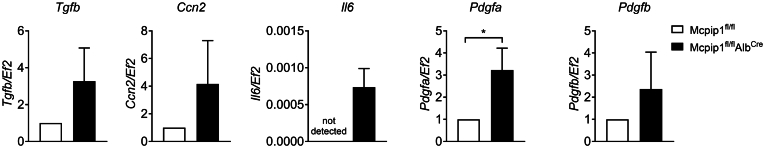
**Expression of profibrotic genes by cholangiocytes.** Expression of *Tgfb1*, *Ccn2, Il6, Pdgfa,* and *Pdgfb* in cholangiocytes from Mcpip1^fl/fl^ or Mcpip1^fl/fl^Alb^Cre^ mice co-cultured with HSCs, n = 3. Graphs show means + SEMs; ∗*P* < .05.

In the livers of Mcpip1^fl/fl^Alb^Cre^ mice, deletion of the *Zc3h12a* gene occurred in both cholangiocytes and hepatocytes (Figure 10*B* and *E*). To elucidate whether cholangiocytes or hepatocytes drive the activation of HSCs in this murine model, we cultured primary HSCs isolated from C57BL6/J mice in the presence of hepatocytes or cholangiocytes isolated from either control or Mcpip1^fl/fl^Alb^Cre^ mice cultured on Transwell inserts (Figures 10*A* and *C* and 3*C*). We found that co-culture of HSCs with cholangiocytes from Mcpip1^fl/fl^Alb^Cre^ mice did not impact the expression of genes coding for markers of HSC activation (*Acta2, Tgfb1, Col1a1, Ccn2*), even though the expression of profibrotic genes by cholangiocytes (eg, *Il6, Pdgfa*) was increased (Figures 10*C* and 11). However, co-culture of HSCs with hepatocytes from Mcpip1^fl/fl^Alb^Cre^ mice resulted in increased expression of *Acta2, Col1a1,* and *Ccn2,* which may indicate activation of HSCs (Figure 10*F*). Next, the transcriptome of primary hepatocytes isolated from 6-week-old Mcpip1^fl/fl^Alb^Cre^ and Mcpip1^fl/fl^ mice^13^ was reanalyzed, and GO term enrichment analysis was performed. We found that genes coding for proteins involved in wound healing, ECM organization and secretion, collagen fibril organization, and regulation of collagen biosynthetic process were up-regulated in hepatocytes from Mcpip1^fl/fl^Alb^Cre^ mice (Figure 10*G*). Among these genes, the expression of *Ccn2*, encoding profibrogenic Ctgf protein, was increased in hepatocytes from Mcpip1^fl/fl^Alb^Cre^ mice (Figure 10*H* and *I*), which may indicate enhanced paracrine activation of HSCs by hepatocytes deprived of Mcpip1 protein.

**Figure 12:**
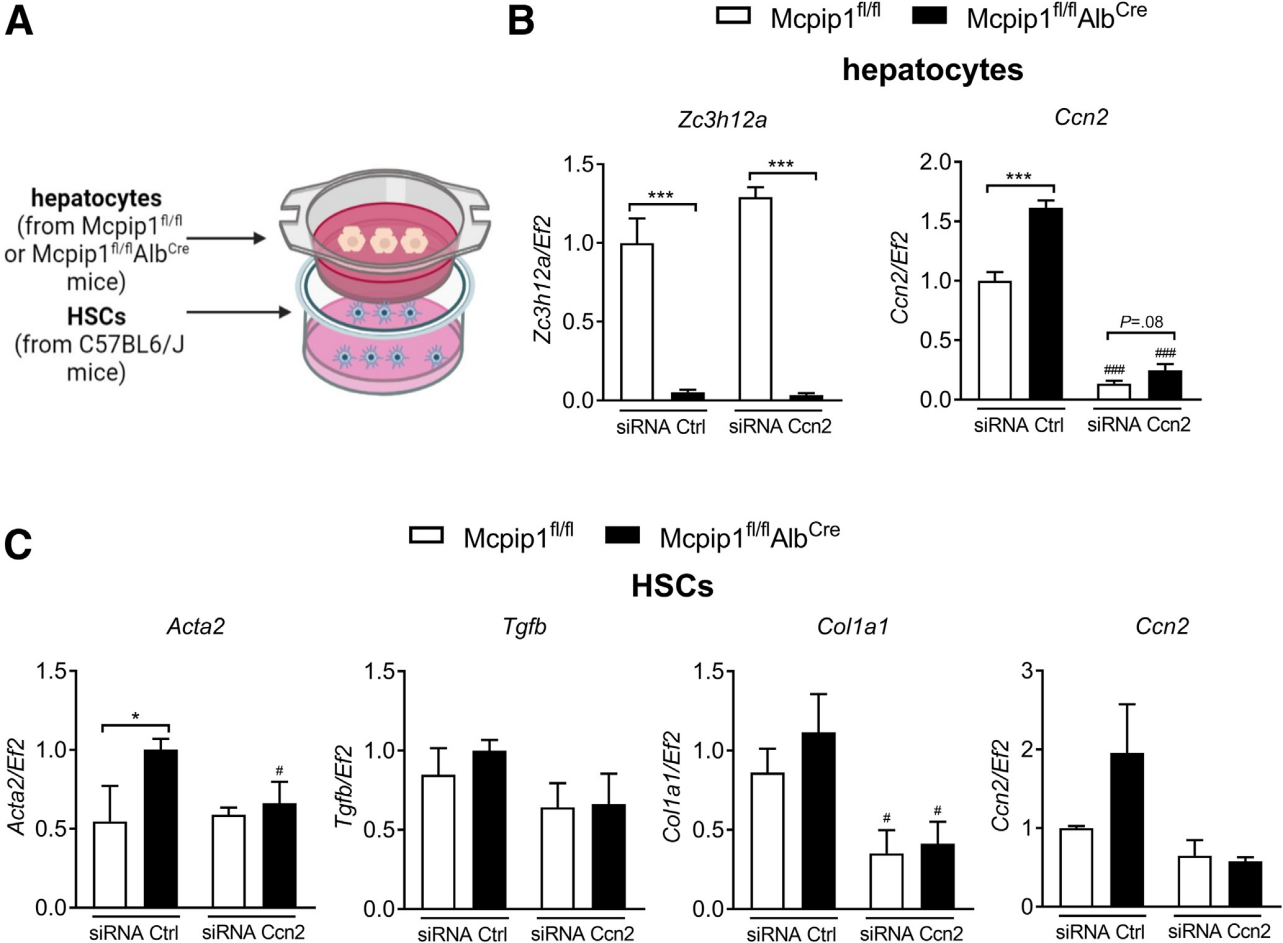
**Silencing of *Ccn2* in Mcpip1**^**fl/fl**^**Alb**^**Cre**^**hepatocytes reduces activation of HCSs.** (*A*) Schematic representation of hepatocyte and HSCs co-culture in Transwell inserts. (*B*) Expression of *Zc3h12a* and *Ccn2* genes in hepatocytes transfected with control (siRNA Ctrl) or Ccn2 smart pool siRNA (siRNA Ccn2). (*C*) Expression of *Acta2, Tgfb1, Col1a1,* and *Ccn2* in HSCs co-cultured with hepatocytes from Mcpip1^fl/fl^ or Mcpip1^fl/fl^Alb^Cre^ mice, n = 3. Graphs show means + SEMs; ∗*P* < .05; ∗∗∗*P* < .001 Mcpip1^fl/fl^ vs Mcpip1^fl/fl^Alb^Cre^; #*P* < .05; ###*P* < .001 siRNA Ctrl vs siRNA Ccn2. Created with BioRender.com.

In the final set of experiments, *Ccn2* gene expression was silenced with small interfering RNA (siRNA) in primary hepatocytes (reduction by 85%–88%) collected from Mcpip1^fl/fl^ and Mcpip1^fl/fl^Alb^Cre^ mice before culturing them with HSCs (Figure 12). Silencing of *Ccn2* expression in primary hepatocytes from Mcpip1^fl/fl^Alb^Cre^ mice reduced activation of HSCs co-cultured with them for 24 hours in comparison with siRNA Ctrl treated cells. Such HSCs had significantly reduced expression of *Acta2* and *Col1a1* in comparison with siRNA Ctrl cells. In addition, there were no differences in HSC activation markers in cells co-cultured with siRNA *Ccn2* transfected hepatocytes isolated from Mcpip1^fl/fl^ and Mcpip1^fl/fl^Alb^Cre^ mice (Figure 12).

## Discussion

TGF-β is a key cytokine driving HSC activation and the development of liver fibrosis. In a quiescent state, HSCs reside in the space of Disse, interposed between LSECs and hepatocytes.^1^^,^^6^ During chronic liver injury, HSCs activate and transdifferentiate into myofibroblast-like cells, along with the acquisition of proinflammatory and profibrogenic properties. Activated HSCs migrate and accumulate at the sites of liver injury, where they secrete large amounts of ECM proteins and regulate their degradation.^1^ If the injury is sustained, accumulated ECM replaces damaged parenchymal cells, forming scar tissue, which disturbs liver architecture and can further result in organ dysfunction.^7^ An increase in TGF-β levels in the process of HSC activation is important for the proper expression of genes coding type one collagen and α-SMA via the SMAD 2/3/4 pathway.^24^, ^25^, ^26^

In the current study, we demonstrated that MCPIP1 expression is induced by the profibrogenic cytokine TGF-β in the human HSC LX-2 cell line. Likewise, it was previously shown that TGF-β–mediated induction of MCPIP1 is observed in podocytes^27^ and the A431 cell line (human SCC cells) on TGF-β treatment.^28^ In addition, induction of epithelial-mesenchymal transition in peritoneal mesothelial cells by TGF-β results in up-regulation of *ZC3H12A* expression, accompanied by increased α-SMA protein levels.^29^ Similarly, on TGF-β stimulation of epithelial-mesenchymal transition in the renal epithelial RPTEC/TERT1 cell line for 48 hours, MCPIP1 is induced [Judyta Go´rka, personal communication].

There is also some evidence of a negative loop in the TGF-β/MCPIP1 axis, as in various models, MCPIP1 expression reduces TGF-β levels. Ectopic expression of MCPIP1 in human proximal renal tubular epithelial cells protects these cells from the effects of TGF-β stimulation.^30^ Another study demonstrated that MCPIP1 impacts TGF-β signaling in breast cancer cell lines by inhibiting the expression of genes coding for members of the TGF-β pathway and therefore suppresses cell migration and metastasis.^31^ Moreover, in Mcpip1^EKO^ mice with epidermal *Zc3h12a* deletion*,* chemically induced squamous cell carcinoma is characterized by enhanced expression of *Tgfb1*.^28^ Our results prove that the overexpression of MCPIP1 in LX-2 cells decreases the expression of *TGFB1, ACTA2,* and *COL1A1* and therefore α-SMA protein levels. In contrast, MCPIP1 silencing reversed this phenotype and led to enhanced HSC activation. Thus, inhibitory action of MCPIP1 on HSCs activation might be at least partially mediated via its RNase activity and direct degradation of *TGFB1* mRNA. Degradation of transcripts by MCPIP1 endoribonuclease activity is mediated by cleavage of hairpin structures. We were able to in silico identify 3 stem‒loop structures in the *TGFB1* mRNA and demonstrate in vitro that MCPIP1_D141N_-Fc can bind to *TGFB1* mRNA and presumably digest it. As already described, MCPIP1 can recognize stem‒loops located both in 3´-untranslated regions (UTRs) and in the coding sequence of mRNAs, eg, in IL-1β, IL-6, TET, and IL-17RA transcripts.^9^^,^^23^^,^^32^^,^^33^

Interestingly, we demonstrated that MCPIP1 can regulate HSC activation not only in a cell-intrinsic but also in a cell-extrinsic manner. For this purpose, we analyzed primary hepatic cells from Mcpip1^fl/fl^Alb^Cre^ mice characterized by a lack of functional Mcpip1 in both hepatocytes and cholangiocytes, which develop substantial liver fibrosis.^13^ To elucidate whether hepatocytes or cholangiocytes impact HSC activation in a paracrine manner in this model, we co-cultured HSCs isolated from C57BL6/J mice with hepatocytes or cholangiocytes from Mcpip1^fl/fl^Alb^Cre^ mice seeded on Transwell inserts. HSCs co-cultured with hepatocytes deprived of Mcpip1 were characterized by increased expression of *Acta2, Ccn2,* and *Col1a1*. In these hepatocytes, expression of *Ccn2*, coding for the profibrogenic mediator Ctgf, was also increased. Ctgf is a protein produced by several cell types that regulates cell adhesion, migration, differentiation, apoptosis, and ECM synthesis in an autocrine or paracrine manner.^34^ Ctgf is overexpressed in numerous fibrotic tissues including the liver, and among its distinct cell types, cholangiocytes, hepatocytes, and HSCs are the main sources of this protein.^34^ Importantly, several studies have demonstrated that hepatocyte-derived Ctgf can stimulate HSCs activation in a paracrine manner.^35^^,^^36^

An interplay between Mcpip1 and Ctgf might be partially mediated by peroxisome proliferator-activated receptor γ (PPARγ). Fu et al^37^ demonstrated, that activation of PPARγ in human aortic smooth muscle cells significantly inhibited TGF-β–induced CTGF production. Mechanistically, PPARγ activation inhibited TGF-β–induced CTGF promoter activity in a dose-dependent manner by directly interfering with the Smad3 signaling pathway. The same effect of PPARγ on TGF-β1–induced CTGF expression was also observed in rat hepatic stellate cells and cat corneal fibroblasts.^38^^,^^39^ On the other hand, MCPIP1 was shown to activate PPARγ in HepG2 cells.^40^ Thus, MCPIP1 knockout hepatocytes might be characterized by lower activity of PPARγ resulting in enhanced *Ccn2* expression and higher Ctgf production. This evidence supports our findings that HSCs activation in Mcpip1^fl/fl^Alb^Cre^ mice may depend on Ctgf produced by hepatocytes lacking the Mcpip1 protein.

Data from in vivo models also show that HSC activation, which occurs along with the development of liver fibrosis, is accompanied by an increase in Mcpip1 levels. We were able to confirm that both in CCl_4_-treated mice and in Mcpip1^fl/fl^Alb^Cre^ animals, Mcpip1 levels are induced in HSCs. Moreover, we determined that MCPIP1 levels are higher in the livers of patients with fibrosis than in those of control individuals. In addition, analysis of RNA sequencing databases confirmed that *ZC3H12A* expression increases in the fibrotic livers of NASH and AH patients. As verified by us in animal models of fibrosis, the increase in MCPIP1 in human fibrotic livers may result from its increase in hepatocytes and HSCs.

## Conclusion

Our study demonstrated that MCPIP1 is induced in human fibrotic livers. In addition, evidence from murine models of liver fibrosis suggests that Mcpip1 expression is increased in hepatocytes and HSCs. We also proved that MCPIP1 can inhibit the activation of HSCs in both autocrine and paracrine manner, playing an important role in the development of hepatic fibrosis.

## Materials and Methods

### Patients

For analysis of MCPIP1 protein levels, samples were collected from 32 patients in the Second Department of General Surgery, Jagiellonian University Medical College (Krakow, Poland). Exclusion criteria included hepatitis C virus/hepatitis B virus/human immunodeficiency virus infection, autoimmune diseases, cancer, and alcohol abuse. Liver samples were isolated during bariatric surgery; one sample of the liver was placed in formalin (for histologic analysis), and the second sample of liver was snap-frozen in liquid nitrogen and then stored at –80°C (for protein analysis). All human tissue samples were collected according to the established protocol approved by the Local Ethics Committee (approval no. 122.6120.263.2016).

For analysis of *ZC3H12A* expression, transcriptomic high-throughput data were downloaded from SRA in fastq format using *SRAtoolkit* version 3.0.0 in a custom script to ensure the correct download of all samples. For RNA sequencing, state-of-the-art processing and analysis pipelines have been set up by the team.^41^ Sequences were trimmed using *TrimGalore* version 0.6.0 with *Cutadapt* version 1.18.^42^ The mapping step was carried out using *STAR* version 020201^43^ over genome version hg38. Read counting was performed using *HTseq* version 0.11.0^44^ and normalized using *EdgeR* version 3.28.1^45^ in R version 3.6.3. The trimmed mean of M-values (TMM) was selected as the method for normalization in addition to low expression gene filtering for differential gene expression analysis. Detailed information concerning the selection of liver samples from patients was provided previously.^20^, ^21^, ^22^

### Animals and Genotyping

Animals were housed under specific pathogen-free conditions in ventilated cages in a temperature-controlled environment with 14-/10-hour light/dark cycle. Liver fibrosis in C57BL/6J male mice was induced by intraperitoneal administration of CCl_4_ (Sigma-Aldrich) (0.6 μL of CCl_4_ per gram of body weight) diluted in corn oil (Sigma-Aldrich) (50 μL per mouse) 3 times a week for 4 weeks, starting from the age of 8 or 9 weeks. For liver epithelial cell-specific knockout of the *Zc3h12a* gene, Zc3h12a^lox/lox^ mice (Mcpip1^fl/fl^), with loxp sites flanking exon 3 of the *Zc3h12a* gene,^46^ were crossed with liver-specific Cre-expressing transgenic mice (Alb^Cre^ tg/+, Jackson Laboratory). The mice were designated Mcpip1^fl/fl^Alb^Cre^. For genotyping, DNA was extracted from tail tissue using the KAPA Mouse Genotyping Kit (KAPA Biosystems) according to the manufacturer’s instructions. Genotyping for loxp insertion was performed by PCR using the following primers: GCCTTCCTGATCCTATTGGAG (wild-type), GAGATGGCGCAGCGCAATTAAT (knockout), and GCCTCTTGTCACTCCCTCCTCC (common). Genotyping for AlbCre tg/+ was conducted with the following primers: TGCAAACATCACATGCACAC (wild-type), GAAGCAGAAGCTTAGGAAGATGG (mutant), and TTGGCCCCTTACCATAACTG (common).

All animal procedures were conducted in accordance with the Guide for the Care and Use of Laboratory Animals (Directive 2010/63/EU of the European Parliament) and carried out under a license from the 2nd Local Institutional Animal Care and Use Committee in Kraków (study no. 82/2020, 74/2021, and 127/2022).

### Histologic Analysis

Liver samples obtained from patients and mice were fixed in 4% buffered formalin and processed through the standard paraffin embedding method. Sections of 10 μm were stained with H&E for general histology and picrosirius red (PSR) for collagen deposition and then visualized using a standard light microscope under ×100 magnification (Leica DMC5400, Leica Microsystems). For quantitative analysis of fibrosis, PSR-stained sections were imaged under ×4 magnification using Leica Application Suite X (LAS X) image acquisition software, and the degree of collagen deposition was analyzed using ImageJ software.

### Isolation and Culture of Primary Hepatocytes

Primary hepatocytes were isolated from murine livers via collagenase perfusion and cultured as described previously.^40^ After isolation, cells were harvested for Western blot analysis or seeded for experiments.

### Isolation of Primary Hepatic Stellate Cells

Primary HSCs were isolated from murine livers via pronase-collagenase perfusion as described previously.^47^ Briefly, animals were anesthetized with ketamine (100 mg/kg) and xylazine (10 mg/kg) administered intraperitoneally. Next, livers were perfused via the inferior vena cava with EGTA solution for 2 minutes, followed by 5 minutes of perfusion with pronase solution and finally 7 minutes of perfusion with collagenase solution. After the liver was excised, it was digested in a Petri dish, transferred into the prewarmed pronase/collagenase solution containing 1% DNase, and stirred at 40°C for 25 minutes. After digestion, the cell suspension was filtered through a 70-μm cell strainer, centrifuged (580*g*, 10 minutes, 4°C), and washed twice with Gey’s balanced salt solution. Then, the cell suspension in Gey’s balanced salt solution was mixed with Nycodenz solution, transferred to four 15-mL Falcon tubes (12 mL to each tube), overlaid with 1.5 mL of Gey’s balanced salt solution, and centrifuged (1380*g*, 17 minutes, 4°C, without brakes). After Nycodenz gradient centrifugation, a thin white layer of HSCs in the interface between the cell-Nycodenz solution and the overlay with Gey’s balanced salt solution was collected and washed with Gey’s balanced salt solution. After isolation, cells were harvested for Western blot analysis and staining or seeded for experiments. Cells were plated in Dulbecco modified Eagle medium (DMEM) (Lonza) with 10% fetal bovine serum (Sigma-Aldrich) and 1% penicillin/streptomycin (P/S) (Lonza) and maintained at 37°C in a humidified atmosphere with 5% CO_2_.

### Isolation and Culture of Primary Kupffer Cells

Primary Kupffer cells were isolated from murine livers via collagenase perfusion as described previously.^48^ Briefly, animals were anesthetized with ketamine (100 mg/kg) and xylazine (10 mg/kg) administered intraperitoneally. Next, livers were perfused via the inferior vena cava with 20 mL of Krebs-Ringer buffer with 0.1 mmol/L EDTA, followed by 30 mL of digestion solution (Krebs-Ringer with 4.76 mmol/L CaCl_2_ and 200 U/mL collagenase IV [Gibco]). After the liver was excised, it was digested in a Petri dish containing 10 mL of complete medium and filtered through a 100-μm cell strainer. The flow-through was centrifuged (50*g*, 3 minutes, 4°C), and the supernatant was collected, layered with a Percoll gradient (25% Percoll underlaid with 50% Percoll), and centrifuged (1200*g*, 30 minutes, 4°C, without brakes). Then, the white cell ring in the middle interphase was collected and washed with phosphate-buffered saline. After that, the cells were resuspended in culture medium (RPMI with 5% fetal bovine serum and 1% P/S), plated for 30 minutes at 37°C in a humidified atmosphere with 5% CO_2_, and then the adherent cells were harvested for Western blot analysis and staining.

### Isolation and Culture of Cholangiocytes

Primary cholangiocytes were isolated from murine livers via collagenase perfusion. Animals were anesthetized with ketamine (100 mg/kg) and xylazine (10 mg/kg) administered intraperitoneally. Next, livers were perfused via the inferior vena cava with 75 mL of wash buffer (0.1 mol/L HEPES, 2.7 mmol/L KCl, 135 mmol/L NaCl, 250 μmol/L HNa_2_PO_4_) with EGTA, followed by 50 mL of digestion solution (HEPES buffer with 10 mmol/L CaCl_2_ and collagenase IV [Gibco]). Next, isolation was performed as previously described.^49^ Briefly, after the liver was excised, it was digested in a Petri dish containing 10 mL of complete medium to wash out hepatocytes, and the remaining biliary tree was digested for 45 minutes in a shaker bath at 37°C with 25 mL of DMEM/F12 with 3% fetal bovine serum, 1% P/S, 25 mg of bovine serum albumin, 6.25 mg of collagenase IV, 8.5 mg of pronase (Sigma-Aldrich), and 1.5 mg of DNase (Roche). Digested tissue was sequentially filtered through 100- and 40-μm filters and then digested for 45 minutes in a shaker bath at 37°C (above 100 μm fraction) or for 30 minutes at 37°C without shaking (fraction between 40 μm and 100 μm) with 25 mL of DMEM/F12 with 3% fetal bovine serum, 1% P/S, 25 mg of bovine serum albumin, 6.25 mg of collagenase IV, 6.5 mg of hyaluronidase (Sigma-Aldrich), and 1.5 mg of DNase. After a second series of filtration through a 40-μm filter, cells were resuspended in fully supplemented DMEM/F-12 medium and seeded on rat tail collagen-coated flasks. Cells were cultured in DMEM/F12 (Lonza) medium with 10% fetal bovine serum, 1% of MEM non-essential amino acids solution (Gibco), 1% of insulin-transferrin-sodium selenite solution (Sigma-Aldrich), 1% of chemically defined lipid concentrate (Gibco), 1% of soybean trypsin inhibitor (Gibco), 1% of L-glutamine (Lonza), 1% of P/S (Lonza), 1% of MEM vitamin solution (Gibco), 0.2% of gentamicin (Gibco), 3.44 μg/mL of triiodothyronine (Sigma-Aldrich), 25 ng/mL of epidermal growth factor (Sigma-Aldrich), 0.4 μg/mL of forskolin (Sigma-Aldrich), 0.4 μg/mL of dexamethasone (Sigma-Aldrich), and 0.12% of bovine pituitary extract and maintained at 37°C in a humidified atmosphere with 5% CO_2_.

### Immunofluorescence Staining

Cells were plated on glass coverslips in 24-well culture plates at a density of 50,000 cells per well. Coverslip cultures were fixed in 4% paraformaldehyde after 24 hours, permeabilized with 1% Triton X-100 in phosphate-buffered saline, and blocked with 0.2% Triton X-100 in 10% goat serum in phosphate-buffered saline. Samples with the following primary antibodies were incubated at 4°C overnight: for HSC staining, rabbit anti-α-SMA (1:100, Abcam), for Kupffer cell staining, mouse anti-CD68 (1:50; Abcam), and for cholangiocyte staining, rat anti-CK19 (1:250, Merck). On the next day, the following secondary antibodies were added for 2 hours in the dark at room temperature: Alexa Fluor 546–conjugated goat anti-rabbit (Thermo Fisher Scientific, diluted 1:500), Alexa Fluor 546–conjugated goat anti-mouse (Thermo Fisher Scientific, diluted 1:500), or Alexa Fluor 594–conjugated goat anti-rat (Thermo Fisher Scientific, diluted 1:500) + Hoechst (Invitrogen, diluted 1:2000) in 5% bovine serum albumin in phosphate-buffered saline. Slides were mounted with glass coverslips using 10 μL of mounting medium (ProLong Gold Antifade) and sealed with nail polish. A Leica DMC5400 fluorescence microscope (Leica Microsystems) with Leica LASX image acquisition software was used to visualize slides.

### Co-Culture

HSCs isolated from C57BL6/J mice were seeded in 24-well plates (150,000/well) and cultured for 24 hours. Then, HSCs were co-cultured with primary hepatocytes (50,000/insert) or cholangiocytes (50,000/insert) isolated from Mcpip1^fl/fl^ or Mcpip1^fl/fl^Alb^Cre^ mice and loaded onto the upper chamber of Transwell inserts with a 0.4 μm pore size (Corning). After 24 hours of co-culture, cells were harvested for real-time PCR analysis.

### Protein-RNA Immunoprecipitation

To perform RNA immunoprecipitation of MCPIP1_D141N_ in a complex with bound RNA, we cloned the coding sequence of human MCPIP1_D141N_ (NM_025079.3) to Fc vector (Addgene number 141183). Insert of MCPIP1_D141N_ was amplified by PCR using primers containing restriction sites for NheI and BamHI. Sequences of primers used for PCR are Forward: 5′-TAGCTAGCGAGTCTGAGCTATGAGTGGC-3′; Reverse: 5′-TAGGATCCTCCTCCCTCACTGGGGTGCTGGGAC-3′. Subsequently, using T4 ligase (New England Biolabs) the coding sequence of MCPIP1_D141N_ was ligated with Fc plasmid. As the result of molecular cloning the coding sequence of MCPIP1_D141N_ has been fused at C-terminus with Fc tag; the polypeptide chain of MCPIP1_D141N_-Fc contains 830 amino acids with molecular weight 91.5 kDa. To isolate complexes of MCPIP1_D141N_-Fc with RNA we used HEK293 cells that were cultured at 37°C in 5% CO_2_ enriched atmosphere in DMEM (Lonza) containing 4.5 g/mL glucose and 10% fetal bovine serum. Cells were plated on 10-cm dish and on the next day were co-transfected with 10 μg of plasmids using jetPRIME transfection reagent (Polyplus) according to manufacturer’s protocol. Control cells were transfected with 8 μg of empty-pcDNA3.0 and 2 μg of TGFB1_pLX307 (Addgene number 98377) plasmids. D141N samples were transfected with 8 μg of MCPIP1_D141N_-Fc and 2 μg of TGFB1_pLX307 plasmids. Cells were lysed 24 hours after transfection using 1 mL low-salt Lysis Buffer containing 20 mmol/L Tris HCl, pH 7.5, 15 mmol/L NaCl, 10 mmol/L EDTA, 0.5% NP40, 0.1% Triton X-100, supplemented with 1 mmol/L PMSF, 1× protease inhibitor cocktail (P8340, Sigma-Aldrich), and 0.1 U/μL SUPERase RNase inhibitor (Invitrogen). After addition of Lysis Buffer, cells were incubated 30 minutes on ice, and subsequently immunoprecipitations of MCPIP1_D141N_-Fc were performed as follows. First, cell lysates were centrifuged for 10 minutes at 17,000 rcf at 4°C, as next soluble fraction of samples was diluted 3-fold using Wash Buffer (20 mmol/L Tris HCl, pH 7.5, 300 mmol/L NaCl, 0.1% NP-40 supplemented with 1 mmol/L PMSF, 1× protease inhibitor cocktail (P8340), and 0.1 U/μL SUPERase). For immobilization of Fc tagged MCPIP1_D141N_ we used Dynabeads G (Invitrogen) magnetic resin. During immunoprecipitation samples were incubated with Dynabeads G for 2 hours at 4°C with gentle rotation. After incubation the flow-through fraction was collected, and the magnetic beads were washed at first using Wash Buffer, followed by 2 washes using Isotonic Wash Buffer (20 mmol/L Tris-HCl, pH 7.5, 150 mmol/L NaCl, 0.1% NP-40). Complexes of MCPIP1_D141N_-Fc with RNA were eluted using 100 μL of acidic Elution Buffer (200 mmol/L glycine, pH 2.5 supplemented with 0.1 U/μL SUPERase); immediately after elution samples were neutralized using 10 μL of 1 mol/L Tris pH 10.4 buffer. Subsequently, 90% of eluted samples were designated for RNA isolation and 10% for Western blotting. RNA bound to MCPIP1_D141N_-Fc was extracted using Fenozol (A&A Biotechnology) as described below.

### LX-2 Cell Culture

The LX-2 cell line was a gift from Dr Matteo Tardelli (Medical University of Vienna)^50^ and was cultured in DMEM with 4.5 g/L glucose with 5% fetal bovine serum, L-glutamine (2 mmol/L), and antibiotics. Cells were cultured at 37°C in a 5% CO_2_ humidified atmosphere and were routinely validated to be mycoplasma free by PCR. For activation, cells were stimulated with 5 ng/mL or 10 ng/mL TGF-β (Cell Signaling).

### Transfection of Primary Hepatocytes

Primary hepatocytes were isolated from Mcpip1^fl/fl^ and Mcpip1^fl/fl^Alb^Cre^ mice, and 50,000 of cells were seeded on Transwell inserts with 0.4 μm pores (Corning). Twenty-four hours later *Ccn2* level was modified by transfecting cells with siRNA (siGENOME Mouse Ccn2 siRNA SMARTpool or control siRNA from Horizon). SiRNA sequences used in the study are listed in Table 1. For transfection 25 pmol of siRNA and INTERFERin (Polyplus) were used. In parallel HSCs were isolated from C57BL/6J mice and plated on 12-well plates. On the following day (24 hours after siRNA transfection) inserts with hepatocytes were transferred to plates with HSCs and co-cultured for 24 hours. Next, RNA from hepatocytes and HSCs were isolated as described below.

**Table 1:** Sequences of siRNA Used for Transfection of Primary Hepatocytes

Name	Catalog no.	Sequence no.	Sequence
siGENOME Non-Targeting siRNA Pool #1	D-001206-13-15	1	UAGCGACUAAACACAUCAA
		2	UAAGGCUAUGAAGAGAUAC
		3	AUGUAUUGGCCUGUAUUAG
		4	AUGAACGUGAAUUGCUCAA
siGENOME SMARTpool siRNA, Ctgf	D-040018-01	1	GAGGAACUAUCCCACCAAA
	D-040018-02	2	GUGAGAACGUUAUGUCAUG
	D-040018-03	3	CAAAGCAGCUGCAAAUACC
	D-040018-04	4	CCAUACAAGUAGUCUGUCA

### Lentiviral Transduction

For determination of the effect of MCPIP1 in the LX-2 cell line, different viral vectors were used as described previously.^40^

### RNA Isolation and Real-time PCR

Total RNA from livers, primary hepatocytes, HSCs, cholangiocytes, and LX-2 cells were isolated using Fenozol (A&A Biotechnology). A NanoDrop 1000 spectrophotometer (Thermo Fisher Scientific) was used to assess RNA concentration and quality. For reverse transcription, 1 μg of total RNA, oligo(dT) 15 primer (Promega), and M-MLV reverse transcriptase (Promega) were used. Real-time PCR was carried out using SYBR Green Master Mix (A&A Biotechnology) and a QuantStudio Real-Time PCR System (Applied Biosystems). Human gene expression was normalized to 18S rRNA, murine gene expression was normalized to Ef2, and then the relative transcript level was quantified by the 2ˆdeltaCt method. Primer sequences (Genomed/Sigma) are listed in Table 2.

**Table 2:** Sequences of Primers Used for Real-time PCR

Gene	Species	Sequence
*Ef2*	Mus musculus, Homo sapiens	F: GACATCACCAAGGGTGTGCAG R: TCAGCACACTGGCATAGAGGC
*Pdgfb*	Mus musculus	F: CCGGAGTCGGCATGAATCG R: TCAAAGGAGCGGATGGAGTG
*Pdgfrb*	Mus musculus	F: GACAGCCAGAAGTAGCGAGAA R: TCACCGTATCGGCAGTATTCC
*Pdgfra*	Mus musculus	F: ATTAAGCCGGTCCCAACCTG R: AATGGGACCTGACTTGGTGC
*Ccn2*	Mus musculus	F: GAGGAAAACATTAAGAAGGGC R: AGAAAGCTCAAACTTGACAG
*Mapk7*	Mus musculus	F: GCTGTCCAAGTCTCAGGTGG R: CAAGCCAGTCAGCAAGGAGA
*Tgfb*	Mus musculus	F: GGATACCAACTATTGCTTCAG R: TGTCCAGGCTCCAAATATAG
*Tgfbr1*	Mus musculus	F: CCAAACCACAGAGTAGGCACT R: GCATAGATGTCAGCGCGTTT
*Col1a1*	Mus musculus	F: GACTGGAAGAGCGGAGAGTA R: GTTCGGGCTGATGTACCAGT
*Col1a2*	Mus musculus	F: CCCAGAGTGGAACAGCGATTA R: ATGAGTTCTTCGCTGGGGTG
*Col3a1*	Mus musculus	F: GAAAGAGGATCTGAGGGCTCG R: GGGTGAAAAGCCACCAGACT
*Mmp9*	Mus musculus	F: CTCTGCTGCCCCTTACCAG R: AGCGGTACAAGTATGCCTCTGC
*Mmp12*	Mus musculus	F: ACCAGAGCCACACTATCCCA R: GCCTCACATCATACCTCCAGT
*Zc3h12a*	Mus musculus	F: CAGCCTCGACCAGATGTGCC R: CAGCCGCTCCTCGATGAAGC
*Acta2*	Mus musculus	F: TCCTGACGCTGAAGTATCCG R: GTCATTTTCTCCCGGTTGGC
*Il6*	Mus musculus	F: ACTTCACAAGTCGGAGGCTT R: GGTACTCCAGAAGACCAGAGG
*Pdgfa*	Mus musculus	F: GTTGTAACACCAGCAGCGTC R: ACCTCACATCTGTCTCCTCCTC
*Mmp3*	Mus musculus	F: GAACAGTCTTGGCTCATGCC R: AGCAGCAACCAGGAATAGGT
*Il1b*	Mus musculus	F: ACCCCAAAAGATGAAGGGCT R: ACAGCTTCTCCACAGCCACA
*Tnfa*	Mus musculus	F: AGGCACTCCCCCAAAAGATG R: GCTCCTCCACTTGGTGGTTT
*18S rRNA*	Homo sapiens	F: CCTTTAACGAGGATCCATTGGA R: CGAGCTTTTTAACTGCAGCAACT
*ACTA2*	Homo sapiens	F: CCTGACTGAGCGTGGCTATT R: GCCCATCAGGCAACTCGTAA
*TGFB1*	Homo sapiens	F: GGACATCAACGGGTTCACTAC R: TGAGAAGCAGGAAAGGCCG
*COL1A1*	Homo sapiens	F: TGAAGGGACACAGAGGTTT R: ACCATCATTTCCACGAGCA
*CCN2*	Homo sapiens	F: GGAAGAGAACATTAAGAAGGGCAA R: CGTCAGGGCACTTGAACTCC

### Transcriptome Sequencing

RNA was isolated from murine livers and primary hepatocytes using the mirVana Isolation Kit (Thermo Fisher Scientific). Libraries were prepared using the Ion AmpliSeq Transcriptome Mouse Gene Expression Kit, which covers more than 20,000 mouse RefSeq genes in a single assay and sequenced on 2 chips from the Ion PI Chip Kit v3 using an Ion Proton Sequencer and Ion PI Hi-Q Sequencing 200 chemistry. RNA sample integrity, library preparation, RNA sequencing, and bioinformatic analysis were performed as described previously.^13^ FASTQ raw files are available under Sequence Read Archive (NCBI) accession numbers SRR23292028-SRR23292043.

### Analysis of TGFB1 mRNA Folding

The transcript of *TGFB1* (NM_000660.7) was analyzed to identify thermodynamically stable stem‒loop structures that contain the YRY consensus motif in a single-stranded loop of hairpin RNA. The *TGFB1* transcript was divided into the 5′-UTR, CDS, and 3′-UTR regions that were subsequently folded by ViennaRNA Package 2.0.^51^ Next, visual inspection of the formed RNA structures was performed to find consensus sequences in the loops of hairpins. The identified stem‒loop structures possessing the YRY feature in the loop were depicted using ViennaRNA Package 2.0 with a sequence-specific color scheme.

### Protein Isolation and Western Blot

Liver samples from primary murine liver cells and LX-2 cells were lysed using RIPA buffer (25 mmol/L Tris-HCl, pH 7.6, 150 mmol/L NaCl, 1% sodium deoxycholate, 0.1% sodium dodecyl sulfate) with Complete Protease Inhibitor Cocktail (Roche) and PhosSTOP Phosphatase Inhibitor Cocktail (Roche). A bicinchoninic acid assay was used to assess the protein concentration. Liver samples from patients and mice were lysed in whole cell lysis buffer (62.5 mmol/L Tris–HCl, pH 6.8, 2% sodium dodecyl sulfate, 25% glycerol, 5% β-mercaptoethanol) with Complete Protease Inhibitor Cocktail (Roche, Basel, Switzerland) and PhosSTOP Phosphatase Inhibitor Cocktail (Roche). A NanoDrop 1000 spectrophotometer (Thermo Fisher Scientific) was used to assess the protein concentrations. Then, 50 μg of protein was separated on 10% sodium dodecyl sulfate‒polyacrylamide gel electrophoresis. After wet transfer onto polyvinylidene difluoride membranes (Millipore), the membranes were blocked in 5% skim milk and then incubated with primary antibodies at 4°C overnight. On the following day, the membranes were washed and incubated with secondary antibody for 1 hour at room temperature. Chemiluminescence was detected after 5-minute incubation with ECL Select Western Blotting Detection Reagent (GE Healthcare) in a ChemiDoc chemiluminescence detector (Bio-Rad). The following antibodies were used: rabbit anti-MCPIP1 (1:2000, GeneTex), rabbit anti-α-SMA (1:1000, Abcam), mouse anti-β-actin (1:4000, Sigma), rabbit anti-GAPDH (1:1000, Cell Signaling), peroxidase-conjugated anti-rabbit (1:30,000, Cell Signaling), and peroxidase conjugated anti-mouse (1:20,000, BD). Anti-MCPIP1 antibodies generated in the core facility were provided by Dr Jacek Jura (National Research Institute of Animal Production, Balice, Poland) and used at 1 μg/mL concentration.

### Enzyme-linked Immunosorbent Assay

For evaluation of the level of Tgf-β in the murine liver, a DuoSet enzyme-linked immunosorbent assay was performed (R&D Systems) according to the manufacturer’s instructions. Absorbance was measured at 450 nm with a reference wavelength of 540 nm using a Tecan Spectra Fluor Plus microplate reader.

### Statistical Analysis

The results are expressed as the mean ± standard error of the mean (SEM). One-way analysis of variance with Tukey’s post-test was applied for comparison of multiple groups. The *P* values are marked with asterisks in the charts (∗*P* < .05, ∗∗*P* < .01, ∗∗∗*P* < .001).
